# Evaluating the Effectiveness and Scalability of the World Health Organization MyopiaEd Digital Intervention: Mixed Methods Study

**DOI:** 10.2196/66052

**Published:** 2024-12-16

**Authors:** Yeonsu Lee, Stuart Keel, Sangchul Yoon

**Affiliations:** 1 Center for Global Development Yonsei Institute for Global Health Yonsei University Health System Seoul Republic of Korea; 2 Faculty of Epidemiology and Population Health London School of Hygiene & Tropical Medicine London United Kingdom; 3 Department of Noncommunicable Diseases Vision and Eye Care Programme World Health Organization Geneva Switzerland; 4 Department of Medical Humanities and Social Sciences College of Medicine Yonsei University Seoul Republic of Korea

**Keywords:** World Health Organization, digital intervention, MyopiaEd, behavior change, risk factor, myopia, refractive error, mobile phone

## Abstract

**Background:**

The rapid rise of myopia worldwide, particularly in East and Southeast Asia, has implied environmental influences beyond genetics. To address this growing public health concern, the World Health Organization and International Telecommunication Union launched the MyopiaEd program. South Korea, with its high rates of myopia and smartphone use, presented a suitable context for implementing and evaluating the MyopiaEd program.

**Objective:**

This is the first study to date to evaluate the effectiveness and scalability of the MyopiaEd program in promoting eye health behavior change among parents of children in South Korea.

**Methods:**

Parents of children aged 7 and 8 years were recruited through an open-access website with a recruitment notice distributed to public elementary schools in Gwangju Metropolitan City. Beginning in September 2022, parents received 42 SMS text messages from the MyopiaEd program over 6 months. This digital trial used a mixed methods approach combining both quantitative and qualitative data collection. Pre- and postintervention surveys were used to assess changes in parental knowledge and behavior regarding myopia prevention. Additionally, semistructured interviews were conducted to explore participants’ experiences in depth and receive feedback on program design. Prior to the intervention, the MyopiaEd program design and message libraries were adapted for the Korean context following World Health Organization and International Telecommunication Union guidelines.

**Results:**

A total of 133 parents participated in this study, including 60 parents whose children had myopia and 73 parents whose children did not. Both groups reported high engagement and satisfaction with the program. Significant increases in knowledge about myopia were observed in both groups (*P*<.001). While time spent on near-work activities did not change significantly, parents of children with myopia reported increased outdoor time for their children (*P*=.048). A substantial increase in eye checkups was observed, with 52 (86.7%) out of 60 children with myopia and 50 (68.5%) out of 73 children without myopia receiving eye examinations following the intervention. Qualitative analysis indicated a shift in parents’ attitudes toward outdoor activities, as increased recognition of their benefits prompted positive changes in behavior. However, reducing near-work activities posed challenges due to children’s preference for smartphone use during leisure periods and the demands of after-school academies. The credibility of the institution delivering the program enhanced parental engagement and children’s adoption of healthy behaviors. Messages that corrected common misconceptions about eye health and provided specific behavioral guidance were regarded as impactful elements of the program.

**Conclusions:**

This study demonstrates the MyopiaEd program’s potential as a scalable and innovative digital intervention to reduce myopia risk in children. The program’s effectiveness provides support for broader adoption and offers valuable insights to inform future myopia prevention policies.

## Introduction

### Background

Myopia poses a significant public health concern worldwide, affecting over 2.5 billion people worldwide [1-3]. Uncorrected myopia remains a leading cause of vision impairment and blindness, particularly in children and young adults [4]. East Asia exemplified this challenge, with urban areas in China, Singapore, and Taiwan exhibiting myopia rates exceeding 20% among early primary school children and exceeding 80% in young adults [5-7]. The growing number of young adults with myopia underscores the urgency of addressing this public health issue, given its association with heightened risks of vision impairment and other serious complications later in life [8].

It is known that both genetic and environmental factors play a role in myopia development and progression. The epidemic rise in myopia particularly prevalent among populations of Asian descent had been explained by environmental influences beyond genetics [9-12]. Recent evidence suggests that lifestyle factors play a significant role, with studies reporting that extensive near-work activities such as screen time, reading for long periods, and close work were risk factors for myopia development in children [13-15]. Conversely, increased time spent outdoors had a protective effect [16-18].

Mobile health (mHealth) interventions have emerged as promising tools for promoting healthier behaviors [19]. The ubiquity of smartphones has created a unique opportunity for the use of mobile technology in health care interventions [19-21]. Studies had shown that digital intervention could effectively improve user health outcomes and promote behavior change among diverse populations [22,23]. Based on these findings, the World Health Organization (WHO) and the International Telecommunication Union (ITU) developed the global initiative Be He@lthy, Be Mobile to promote the mHealth interventions for addressing noncommunicable diseases and other health concerns, using modalities including SMS text messages, apps, and chatbots.

Building on the success of Be He@lthy, Be Mobile initiatives in promoting positive health behavior changes and with growing evidence supporting the effectiveness of mHealth interventions, WHO and ITU launched the MyopiaEd program in 2022 [24-27]. This program aims to enhance knowledge and encourage healthy eye habits, ultimately aiming to delay the onset and slow the progression of myopia. mHealth interventions were expected to effectively support eye health behavior modifications, such as increased outdoor time and reduced near-work activities, which are critical for preventing myopia progression, by delivering consistent reminders and prompts [28].

To evaluate the program’s effectiveness and scalability, Yonsei University Health System (YUHS) and Good People International collaborated on a MyopiaEd program implementation in South Korea. South Korea, with its high myopia prevalence and widespread smartphone use, presents a compelling environment for implementing digital interventions [29,30]. This study hypothesizes that the MyopiaEd program will enhance parental knowledge and promote positive behavioral changes in children’s eye health.

### Objective

This study is the first to pilot the MyopiaEd program with a key end-user group, aiming to generate evidence on its effectiveness and scalability. The implementation involves adapting the evidence-based MyopiaEd message libraries and program design to the South Korean context.

## Methods

### Study Design

This study used a mixed methods approach using both quantitative and qualitative data collection methods. Surveys were administered to participants before and after the program to assess changes in knowledge and attitudes related to myopia. After completing the surveys, semistructured interviews were conducted to explore their experiences in more depth. The interview questions were guided by themes identified from the survey responses. To ensure methodological rigor and trustworthiness, the study adhered to the COREQ (Consolidated Criteria for Reporting Qualitative Data) guidelines and the iCHECK-DH (Guidelines and Checklist for Reporting Digital Health Implementations) [31,32].

### Study Setting

#### Step 1: Content Adaptation

The MyopiaEd program design and message libraries were adapted for the Korean context, following WHO and ITU guidance on the adaptation process, to ensure clarity and relevance for the target population [33]. From the suggested target end-user groups, we selected a key population group: parents of children aged between 7 and 8 years to target delaying myopia onset. The MyopiaEd message libraries, covering general myopia knowledge, behavior change, and the importance of regular eye examinations and addressing misconceptions, underwent a comprehensive adaptation process. Two independent researchers (YL and SY) translated the original content into Korean and culturally adapted the messages to align with the Korean context. Subsequently, a review panel consisting of a frontline health worker and a board-certified ophthalmologist conducted a rigorous review of the adapted materials. A reverse translation process, followed by a WHO review, ensured the accurate conveyance of the original meaning in the adapted content. The final message library was established based on the feedback received.

#### Step 2: Pretesting

Pretesting involved 11 participants receiving 14 core MyopiaEd messages over 2 weeks. In-person focus group interviews, conducted by trained interviewers, assessed the understandability and acceptability of individual messages, message format, timing, and channel. This was undertaken to ensure that the program design and message content resonated well with the target audience. Details on key pretesting feedback and the resulting changes are provided in Multimedia Appendix 1.

#### Step 3: Key Revisions Based on Pretesting

Based on pretesting results and feedback, the message libraries underwent key revisions. Messages were revised to include multimedia content, information addressing misconceptions about myopia, and trending Korean eye health issues. Evidence-based visual content was developed in partnership with Weknew Inc, a medical information content company. Finally, an ophthalmologist from the WHO MyopiaEd program’s informal expert group reviewed the message library and multimedia content to ensure clinical accuracy and evidence-based content.

### Recruitment

With the aim of preventing myopia progression in the early stages, parents of children aged 7 and 8 years were selected as the target audience, as myopia rates begin to increase rapidly among Korean children at this age [34]. Participants were recruited through an open-access website of Good People International, with a recruitment notice distributed to public elementary schools located in Gwangju Metropolitan City. These public schools are under the jurisdiction of the Gwangju Metropolitan Office of Education, part of the Ministry of Education. During recruitment, participants were provided with an overview of the study’s purpose, intervention process, and potential benefits, along with instructions for accessing and completing the web-based survey. The institutional review board (IRB)–approved informed consent form was embedded in the initial application form to ensure participants understood the potential implications of study participation. Those who agreed to participate and completed the presurvey were enrolled. To prevent duplicate entries, the contact information was reviewed to verify the uniqueness of each user.

After the 6-month intervention, parents who showed significant improvement in myopia knowledge were selected to participate in a qualitative study to explore the underlying mechanisms driving their behavioral changes. The interviews aimed to understand how receiving MyopiaEd messages increased knowledge about myopia prevention among parents and how this knowledge translated into actual changes in their children’s eye health behaviors.

### Intervention

The program was launched in June 2022 with the primary objective of enhancing knowledge about myopia and promoting good eye health behaviors for the prevention and management of myopia. Following the WHO MyopiaEd toolkit guidelines, a total of 42 adapted messages were sent to the target group over a 6-month period. This intervention was conducted as a digital trial using SMS text messages as the delivery method. Parents received SMS text messages twice a week for 18 weeks. After that, the frequency of messages was reduced to once a week for the remaining 6 weeks. SMS text messages and multimedia messages with images were delivered alternately. For the multimedia messages, the resources were uploaded to the program website and were delivered via messages along with the post link. Messages were sent via KakaoTalk (Kakao Corp), the most widely used messaging app in Korea, with a large and highly engaged user base [35]. The message libraries delivered to parents are included in Multimedia Appendix 2.

### Data Collection and Measurement

#### Overview

This study assessed changes in knowledge and behavior (primary outcomes) following the intervention, along with suggestions for program design and message content (secondary outcomes). Behavior changes were evaluated by considering near-work activities as a risk factor and time spent outdoors, wearing glasses, and eye examination uptake as protective factors. To examine the potential association between behavior change intensity and intervention outcomes, we categorized eye health behaviors into 3 levels: mild, moderate, and intensive. These levels were defined based on time commitment, financial burden, and required effort. Increasing outdoor activity was classified as a mild intervention, while reducing near-work activities and getting eye examinations were categorized as moderate and intensive interventions, respectively.

#### Quantitative Study

Quantitative data were collected using a self-assessment questionnaire developed based on WHO monitoring and evaluation indicators [36]. Surveys were conducted in accordance with the CHERRIES (Checklist for Reporting Results of Internet E-Surveys) as detailed in Multimedia Appendix 3 [37]. In the posttest, participants self-reported their engagement and satisfaction with the program. Satisfaction with the content was measured on a 5-point Likert scale, ranging from 1=strongly disagree to 5=strongly agree. Participants also indicated their perceived changes in knowledge and behavior compared to before the intervention, with options including “improved” and “not improved” for knowledge and “increased,” “not changed,” or “decreased” for behavior.

A survey questionnaire developed by the research team was used to assess participants’ knowledge of myopia in both the pre- and posttests. Participants responded to 16 questions with a yes, no, or unsure response. Correct responses were given a full score of 100, while incorrect or uncertain responses received 0. To assess behavior change, participants self-reported the average daily time spent on outdoor activities and near-work activities during their leisure time on weekdays and weekends, specifying the time in hours and minutes. For parents of children with myopia only, the frequency of wearing prescribed glasses among their children was measured using a 5-point Likert scale (1=never and 5=always).

#### Qualitative Study

Six months after the intervention, semistructured interviews were implemented by a trained interviewer via videoconference using a semistructured interview guide. Interviews lasted approximately 1 hour and were conducted in Korean at a time convenient for the participants. During the interviews, participants were asked to recall memorable messages and provide feedback on individual messages’ impact on behavior change. Among the MyopiaEd messages sent, a core set of 24 messages was specifically discussed. Additionally, participants provided their opinions on program design aspects such as program duration, communication channels, and message frequency. With informed consent, interviews were audio-recorded, and all participant information was documented.

### Statistical Analysis

Quantitative statistical analyses were performed using R software (version 4.2.2; R Foundation for Statistical Computing). Paired-sample 2-tailed *t* tests and Wilcoxon signed rank tests were used to examine changes between pre- and postintervention evaluations. Prior to the analysis, the normality of the data was assessed using the Shapiro-Wilk test. For the qualitative study, common themes were identified using an inductive thematic approach.

### Ethical Considerations

This study was conducted in accordance with the principles of the Declaration of Helsinki. Approval was granted by the IRB, Severance Hospital, Yonsei University, Seoul, Korea (IRB approval 4-2022-0798). All participants provided written informed consent prior to study enrollment and were informed of their right to withdraw at any time. The data were anonymized for privacy and confidentiality. As compensation for completing the pre- and postintervention surveys, participants received a 100,000 KRW (US $76.32) gift certificate for an optical store.

## Results

### Quantitative Study

**Figure 1:**
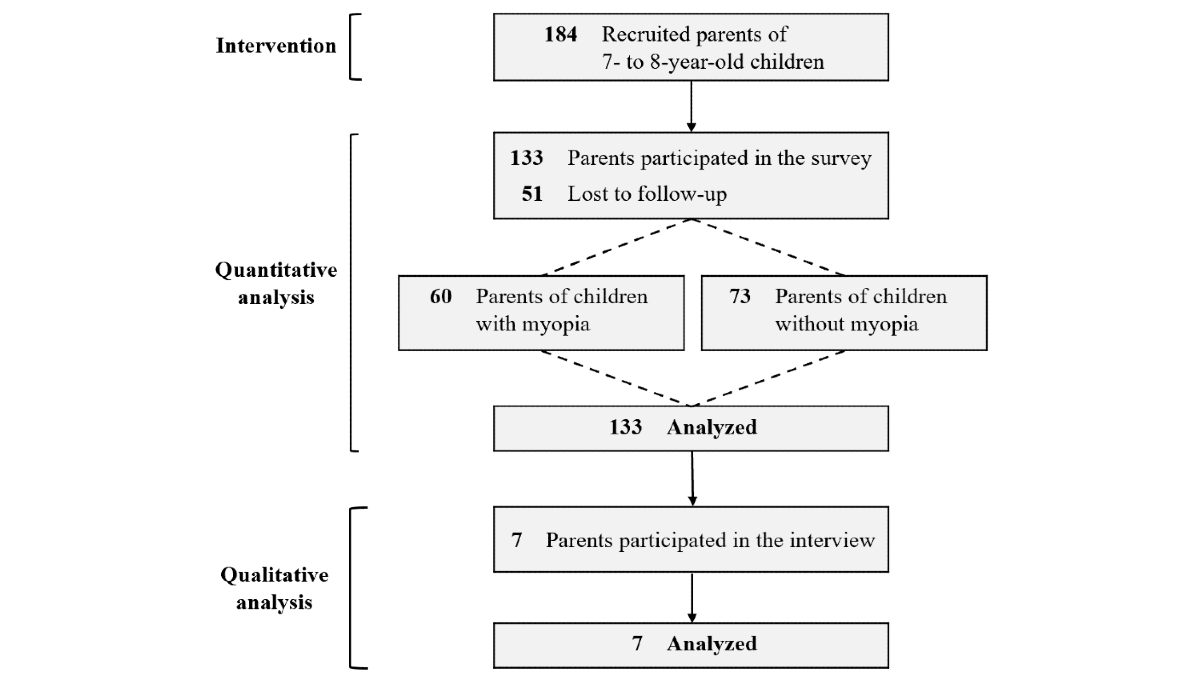
Flow diagram illustrating participant involvement in the intervention, quantitative analysis, and qualitative analysis within the MyopiaEd program.

Figure 1 illustrates the flow diagram of the study participants. From September 2022, a total of 184 parents of children aged 7 to 8 years were recruited for this study. After a 6-month intervention, 133 parents who completed both pre- and postintervention survey were evaluated, consisting of 60 parents of children with myopia and 73 parents of children without myopia. The mean age of parents of children with myopia was 40.38 (SD 2.91) years, and the mean age of parents of children without myopia was 39.52 (SD 3.94) years (Table 1). The parent group was predominantly female, with 56 (93.3%) out of 60 parents of children with myopia and 72 (98.6%) out of 73 parents of children without myopia being female. Parents of children with myopia were more likely to have a personal eye care routine for their children (*P*=.003), including practices like limiting screen time. There was also a significant difference (*P*<.001) in the timing of the last eye examination. In total, a vast majority (54/60, 90%) of parents of children with myopia reported their children having had an eye examination within the past 6 months compared to 32 (43.8%) out of 73 parents of children without myopia. An additional 16 (21.9%) out of 73 parents in the nonmyopic group reported an examination between 6 months and 1 year.

**Table 1:** Demographic characteristics and baseline eye care practices of parents and their children with and without myopia (N=133).

Characteristics			Parents of children with myopia (n=60)		Parents of children without myopia (n=73)		*P* value	
**Age (years), mean (SD)**			40.38 (2.91)		39.52 (3.94)		.12	
**Sex, n (%)**								.17
	Female	56 (93.3)		72 (98.6)				
	Male	4 (6.7)		1 (1.4)				
**Children’s age (years), mean (SD)**			7.65 (0.6)		7.52 (0.6)		.18	
**Children’s sex, n (%)**								.71
	Female	30 (50)		40 (54.8)				
	Male	30 (50)		33 (45.2)				
**Children’s personal eye care routine, n (%)**								.003
	Yes	39 (65)		27 (36.9)				
	No	17 (28.3)		40 (54.7)				
	Do not know	4 (6.7)		6 (8.2)				
**Children’s last eye examination, n (%)**								<.001
	Less than 6 months	54 (90)		32 (43.8)				
	More than 6 months and less than a year	2 (3.3)		16 (21.9)				
	More than a year and 2 years	1 (1.7)		12 (16.4)				
	More than 2 and 5 years	0 (0)		3 (4.11)				
	More than 5 years	0 (0)		0 (0)				
	Do not know	3 (5)		10 (13.7)				

The results of the quantitative study are presented in Tables 2-4. Both parents of children with and without myopia showed high engagement with the program, with no significant difference observed between the 2 groups (Table 2). In total, 38 (63.3%) out of 60 parents of children with myopia and 51 (69.9%) out of 73 parents of children without myopia shared the message content with their families or friends who were not enrolled in the program. Participants rated the program highly across all aspects of content satisfaction, including ease of understanding, applicability, and relevance.

During the 6-month follow-up, participants evaluated their changes in knowledge and children’s behavior related to myopia (Table 3). A vast majority of parents of children with myopia (54/60, 90%) and parents of children without myopia (68/73, 93.2%) subjectively reported an improvement in their knowledge of myopia after participating in the program. Most parents reported that their children spent more time on outdoor activities and less time on near-work activities during leisure periods regardless of the child’s myopia status. However, parents of children with myopia were more likely to complete their children’s eye examinations after the intervention compared to parents of children without myopia (*P*=.02).

Both groups showed statistically significant improvements in their knowledge about myopia (Table 4). Regarding behavior change, parents of children with myopia reported a significant increase in outdoor activity time after the intervention (*P*=.048). While parents of children without myopia also reported increased outdoor activity, this change was not statistically significant (*P*=.06), although it suggests a trend toward significance. There was no significant decrease in the amount of time children spent on near-work activities in either group. Parents of children with myopia reported a significant increase in the frequency of their children wearing prescribed glasses (*P*<.001).

Multimedia Appendix 4 shows the individual scores out of 100 for each of the 16 questions on the knowledge assessment questionnaire used to evaluate participants’ understanding of myopia. Participants in both groups showed improvement in their understanding of several myopia-related topics. Specifically, they gained knowledge about the definition of myopia (question 5), the benefits of outdoor activities (question 9), the importance of wearing glasses (questions 10 and 12), and the role of environmental factors in myopia development (question 4). However, participants’ understanding of the negative impact of close working distance, regardless of activity (reading and smartphone use), did not significantly improve (questions 6 and 8).

**Table 2:** Engagement and satisfaction levels of participating parents with the MyopiaEd program over a 6-month period (N=133).

Variables			Parents of children with myopia (n=60)		Parents of children without myopia (n=73)		*P* value	
**Estimation of the messages read by users, n (%)**								.75
	None	0 (0)		0 (0)				
	Fewer than half	3 (5)		6 (8.2)				
	More than half	18 (30)		23 (31.5)				
	Nearly all	39 (65)		44 (60.3)				
**Users sharing the message content with others not enrolled in the initiative, n (%)**								.37
	Yes	38 (63.3)		51 (69.9)				
	No	22 (36.7)		22 (30.1)				
**Satisfaction with the content, mean (95% CI)**								
	Ease of understanding the messages	4.72 (4.55-4.88)		4.96 (4.91-5.01)		.006		
	Easy to operationalize advice or instructions	4.83 (4.70-4.97)		4.85 (4.73-4.96)		.93		
	Content appropriateness	4.85 (4.74-4.96)		4.85 (4.74-4.96)		.92		
	Mean	4.80 (4.69-4.91)		4.89 (4.81-4.96)		.11		

**Table 3:** Self-reported changes in parental knowledge and children’s behaviors over the 6-month MyopiaEd program (N=133).

Variables			Parents of children with myopia (n=60), n (%)	Parents of children without myopia (n=73), n (%)	*P* value	
**Parental knowledge gain**						
	**Change in knowledge of myopia**					.54
		Improved	54 (90)	68 (93.2)		
		Not improved	6 (10)	5 (6.9)		
**Behavior change in children**						
	**Change in average daily time spent outdoors**					.94
		Increased	40 (66.7)	50 (68.5)		
		Not changed	19 (31.7)	21 (28.8)		
		Decreased	1 (1.7)	2 (2.7)		
	**Change in average time spent on near-work activities during leisure periods**					.53
		Decreased	37 (61.7)	38 (52.1)		
		Not changed	19 (31.7)	30 (41.1)		
		Increased	4 (6.7)	5 (6.9)		
	**Change in daily time wearing their prescribed glasses**					
		Increased	14 (23.3)	—^a^	—	
		Not changed	41 (68.3)	—	—	
		Decreased	5 (8.3)	—	—	
	**Eye examination uptake**					.02
		Yes	52 (86.7)	50 (68.5)		
		No	8 (13.3)	23 (31.5)		

^a^Not applicable.

**Table 4:** Parental knowledge scores and children’s behavioral measurements at baseline and endline in the 6-month MyopiaEd program (N=133).

Variables		Parents of children with myopia (n=60)						Parents of children without myopia (n=73)				
		Baseline, mean (95% CI)		Endline, mean (95% CI)	*P* value		Baseline, mean (95% CI)			Endline, mean (95% CI)		*P* value
**Parental knowledge gains**												
	Knowledge score	75.21 (72.27-78.15)	83.44 (81.54-85.34)		.001	75.68 (72.21-29.16)			84.33 (82.66-86.00)		.001	
**Behavior change in children**												
	Time spent outdoors (minutes per day)	82.88 (67.12-98.64)	97.07 (83.41-110.73)		.048	82.52 (69.80-95.25)			93.27 (82.48-104.05)		.06	
	Time spent on near-work activities during leisure periods (minutes per day)	165.44 (137.65-193.23)	143.75 (124.73-162.77)		.06	154.36 (129.85-178.88)			140.15 (123.02-157.28)		.51	
	Frequency of wearing prescribed glasses, 5-point Likert scale	3.14 (2.67-3.62)	3.64 (3.22-4.07)		<.001	—^a^			—		—	

^a^Not applicable.

### Qualitative Study

#### Overview

In-depth interviews were conducted with a selection of parents who showed an improvement in knowledge scores (Multimedia Appendix 5). All 7 participants were mothers. In total, 57% (4/7) identified increased the time spent outdoors by their children, while 43% (3/7) decreased the time spent on near-work activities by their children.

#### Eye Health Behavior Change

##### Eye Health Checkup and Vision Correction

When parents were asked about changes in their children’s eye health behavior after participating in the program, all participants responded that their children had undergone eye examinations. Additionally, 71% (5/7) mentioned that their children began wearing eyeglasses or orthokeratology lenses following eye health checkups.

I didn’t pay much attention to eye clinics before because everyone in my family had good eyesight, and I didn’t wear glasses...I only visited eye clinics with my children occasionally for minor issues like inflammation...However, I’ve come to realize the importance of regular eye check-ups through the program. Since this year, I’ve been taking my eldest child for check-ups every six months...and [he] started wearing glasses in March. This experience made me more aware.

Parent 6

Before the campaign, my child had vision tests at school and was found to need vision correction. However, both my husband and I felt it might be too early to wear glasses, so we asked the teacher if our child could sit closer to the front [in the classroom] and delay wearing glasses a bit longer...However, upon receiving a message indicating otherwise, we promptly went to [optometrist to] get eyeglasses fitted for my child.

Parent 7

My child’s vision was exceptionally good until first grade...However, due to factors like the COVID-19 pandemic, we couldn’t arrange any check-ups for a year. Then, in second grade, my child began having trouble seeing. Just at that time, we received a timely message reminder from the program, prompting us to realize the importance of not missing the check-up schedule. So, I promptly took my child for an examination and discovered his vision had worsened.

Parent 4

##### Time Spent on Outdoor Activities

Many parents reported that their children tend to spend most of their weekdays indoors due to after-school academies or both parents working, making it difficult to allocate time for outdoor leisure activities. Therefore, they preferred to engage in outdoor activities during weekends such as taking walks in the park or going camping with their children. They also noted constraints in increasing outdoor activity time, such as their children’s frequent use of smartphones during leisure hours. Some parents mentioned that they had previously been aware only of the negative effects of outdoor activities on eye health due to UV exposure. Following the intervention, they developed a better understanding of the importance and benefits of outdoor activities and encouraged their children to spend more time outdoors.

I allowed my child to engage in more outdoor activities because I learned that playing outside under sunlight isn’t necessarily harmful due to UV rays, but rather beneficial for eye health...The information about spending more than 90 minutes outdoors was meaningful...Children spend most of their time indoors, often glued to their phones.

Parent 5

As a dual-income couple, my family primarily spends weekdays indoors, because children attend academies [until the evening after school]...While we do try to get outdoors on weekends such as camping, the overall change in outdoor time might not be very noticeable...However, both the children and I are aware of the benefits of sunlight for eye health. Even though time spent outdoors haven’t significantly increased, perhaps we’ve made a bit more effort to spend extra time in the sun unconsciously.

Parent 4

Outdoor activities were practically impossible during the weekdays. So, I tried to allow my child to engage in longer activities at least on weekends.

Parent 7

##### Time Spent on Near Works

In total, 71% (5/7) of the parents reported that when they advised their children to reduce near-work activities such as reading books or looking at smartphone screens closely, their children perceived it as nagging. Despite these constraints, 2 parents mentioned that their children voluntarily practiced healthy behaviors, attributing the increased credibility of the messages to the reputation of YUHS and WHO.

Even though I told my children to sit farther away from the TV or take breaks, they didn’t seem to listen well as they got older. After joining the program, I directly shared the messages and materials received from the program with them...These messages had a direct impact on the kids...they saw the messages and materials [from program] and realized, “Oh, it was sent from the medical center [YUHS].” So, they trust it more than me...when I say, “Let’s take a moment to look at something green in the distance,” they’re more willing to comply. I also shared these with friends...[and] online community for moms. Rather than gathering information sporadically, getting information from experts and engaging with questions and answers is far more trustworthy. It was reassuring and beneficial.

Parent 4

Previously, I would ask my kids to sit a bit farther away from the television, but I guess they often felt like nagging...messages from the program now support what I used to say verbally, making it more convincing.

Parent 1

#### Feedback of MyopiaEd Message Content

When participants were questioned about the impactful message content, 86% (6/7) highlighted information regarding trending eye-related topics such as orthokeratology and unsubstantial intervention or nutritional support for myopia prevention. All of the participants considered messages that provide numerical guidance for behavioral changes as memorable, including the 20-20-20 rule, engaging in outdoor activities for at least 90 minutes, and scheduling eye health checkups every 6 months. Information on trending eye-related issues is present in Textbox 1. Clear guidance for behavioral change in messages is present in Textbox 2.

**Information on trending eye-related issues.**

“I inquired about Ortho-K lenses [orthokeratology] and received additional information via chat from an eye health professional. Feeling reassured by the expert’s advice, I learned more about Ortho-K lenses and now my first child is wearing them. Concerned about not missing early screenings, I also went for regular check-ups for my second child. Initially hesitant, receiving continuous messages made me realize the importance of not missing the timing. During the campaign, I went two or three times every three months [for my children], and now my second child is wearing glasses” [Parent 4].

“I used to believe that wearing blue light-blocking glasses was obligatory. However, through messages, I learned that the blue light doesn’t have such a significant impact [for the eye]...Even though the optician recommended blue light blocking glasses, my child didn’t choose to get them...These insights [from messages] were also helpful” [Parent 5].

“Growing up, we were always told that carrots and blueberries were good for our eyes. I’ve always told my kids the same thing. So, I was a bit surprised to learn that it’s not actually true and that these foods don’t necessarily improve vision” [Parent 6].

**Clear guidance for behavioral change in messages.**

“Specifying 90 minutes made me realize that my child’s outdoor activity time had been quite limited. When I measured the time, 90 minutes was longer than I had anticipated...[and] practicing regular outdoor activities for over 90 minutes seemed challenging. However, after a few attempts, I found it to be achievable. It inspired me to include outdoor activities more frequently in our routine...” [Parent 3].

“I made a conscious effort to integrate the 20-20-20 rule into our daily routine. This involved encouraging the kids to take a 20-second break every 20 minutes, focusing on an object at least 20 feet (6 meters) away. It proved to be effective, prompting me to consistently remind the kids to take breaks while using electronic devices or reading” [Parent 5].

“Previously, I didn’t prioritize eye check-ups, only visiting the eye clinic occasionally for minor issues like inflammation in the kids’ eyes. However, recognizing the importance of regular check-ups every six months starting this year, I’ve been ensuring that the kids undergo check-ups every six months. This preventive measure seems to be beneficial” [Parent 6].

#### Feedback of MyopiaEd program

##### Two-Way Messaging

A 2-way messaging system was implemented to foster participant engagement, enabling communication and addressing participants’ curiosity. Interviews revealed that this feature was highly valued, providing opportunities for immediate clarification and enhancing the eye health–related behavior change of participants.

I inquired about Ortho-K lenses and received additional information via chat from an eye health professional. Feeling reassured by the expert’s advice, I learned more about Ortho-K lenses and now my first child is wearing them.

Parent 4

I asked a question and got an answer...I asked something like, “If parents have poor eyesight, is there a high chance that their children will also have poor eyesight?”...Since my child was growing up, I became increasingly aware of the importance of their eye health. Therefore, being able to ask questions and get answers from an ophthalmologist was helpful.

Parent 7

##### Message Format and Channel Used

All participants expressed high satisfaction with the channel, highlighting the messaging app’s convenience for confirming and archiving messages. Most parents positively evaluated the provision of website links, which allowed them to access visual content and more detailed information about the message topics if they were interested. However, some mentioned that the SMS text messages were meaningful even without multimedia, as they effectively conveyed the core message directly.

Receiving messages via KakaoTalk felt convenient...Accessing information directly through messages was more convenient than accessing a blog. However, when I wanted more detailed information on certain topics, I visited the blog.

Parent 3

Using KakaoTalk was convenient for me. I could easily check messages and explore more information through the linked blog if needed...Plus, the connection between KakaoTalk and the blog made it flexible. Even if I missed a message, I could still access the blog without opening a separate browser. Overall, I found this setup to be quite convenient.

Parent 5

##### Frequency and Duration of the Program

Participants responded that the 6-month duration of the campaign was appropriate, and some indicated that a longer duration would have been acceptable as well. Regarding the frequency of message delivery, most agreed that once a week was appropriate, while some suggested that once every 2 weeks would also have been suitable.

While it might have felt lengthy...I think it would be beneficial to continue consistently. I believe maintaining periodic communication, rather than limiting it to a few months as a campaign, wouldn’t be a bad idea. I didn’t pay much attention to the frequency, but it felt quite frequent.

Parent 1

With the 6-month campaign ending, I feel like it was a valuable period during which we acquired a lot of useful information. I didn’t find it too short or too long. As for the frequency, once or twice a week felt reasonable.

Parent 2

The 6-month duration seemed appropriate. Usually, campaigns like this end quickly. Each message was a reminder, and even if I confirm late, receiving another message encouraged me not to delay [eye examination]. They discussed the risks, provided explanations on good practices, and even offered counseling. So, even if the messages continued, I might have been able to ask questions whenever I had doubts [about my children’s eye health]...[Regarding frequency,] Perhaps once a week would have been better. If they come too frequently, attention may weaken slightly. Allowing some time and instilling a sense of urgency would be also beneficial.

Parent 4

When the message came announcing the end, I thought, “Already over?” It passed by quicker than I expected. Once a week for message frequency seems appropriate.

Parent 7

## Discussion

### Principal Findings

This is the first study to evaluate the effectiveness and scalability of the WHO’s MyopiaEd program and its message libraries to enhance awareness and encourage behavior changes aimed at preventing myopia. Increased parental knowledge about myopia appears to be associated with positive behavior changes in children. Increased outdoor time and reduced near-work activities were identified in children both with and without myopia. Recognizing the importance of regular eye examinations for myopia prevention, targeted mHealth intervention to parents resulted in a rise in the proportion of children receiving eye examinations.

This study explored the impact of digital interventions targeting parents as a strategy for myopia prevention in children. Given the limitations of direct engagement with children in a digital approach, targeting parents through informative messages presents a promising approach. Children’s successful behavioral change depends on parents’ understanding of myopia and their willingness to adopt the proposed myopia management and treatment [38]. The program’s design appeared to facilitate positive behavioral changes among participating parents regardless of their children’s existing eye health status, as evidenced by high engagement and a significant increase in eye health awareness. Several factors were contributed to this finding. First, parents with myopia or a family history of the condition exhibited concern for their children’s eye health, even if their children do not currently have any vision problems. Second, witnessing the development of myopia in other children heightened parental vigilance toward myopia prevention. These suggest that myopia prevention programs should consider broadening their target audience to include parents of all children within the susceptible age range, especially in high-prevalence regions experiencing a rising incidence of myopia. Early intervention is particularly important, as research has shown that early myopia onset or rapid progression is the most significant predictor of severe myopia in children [39].

A potential association between the intensity of behavior change and the targeted eye health behavior outcome in children was investigated in this study. Increased outdoor activity was hypothesized to be a mild intervention, as parents could easily facilitate it. This aligns with a case study reporting that a messaging app–based mHealth approach increased parental engagement in promoting outdoor activities for their children [40]. Conversely, reductions in near-work activities such as reading and screen time assumed as moderate did not identify statistically significant results. This is due to the challenges posed by South Korea’s societal pressure on academic achievement and the growing issue of children’s dependence on smartphones [41,42]. A qualitative study from China supports this notion, reported the challenges of family-based interventions where parents prioritize immediate academic success over the long-term risks of vision problems in their children [43]. In addition, promoting regular eye examinations was categorized as more intensive due to logistical considerations and cost burdens. However, our study observed unexpectedly high eye examination uptake due to several contributing factors. These factors include the relative affordability of eye examinations in South Korea, enabled by health insurance coverage, and the widespread availability of ophthalmology services within metropolitan areas.

This study targeted parents of primary-level children among the 4 end-user groups for the MyopiaEd program suggested by WHO, as early detection and management in childhood are essential, and parents have a significant impact on their children’s behaviors at the primary level. By focusing on parents, the program aims to leverage their pivotal role in forming lifestyle habits that can slow the onset of childhood myopia and reduce the risk of developing high myopia. Despite efforts to recruit parents without sex bias, cultural norms in South Korea where female parents are primarily responsible for childcare and domestic tasks likely influenced the dominant proportion of female involvement. Given this childcare disparity, it is understandable that female parents were more active in seeking resources such as the MyopiaEd program to support their children [44]. As this study is the first to evaluate the MyopiaEd program, a crucial next step is to expand its implementation to other target end-user groups based on our findings.

The findings from this initial adaptation of the MyopiaEd program yielded valuable lessons learned regarding program design and message libraries. First, 2-way messaging with question-and-answer interactions, addressing participant inquiries and concerns, contributed to increased participant satisfaction and adherence. Second, concise messages with direct behavioral instructions were sufficient for knowledge dissemination and for promoting behavior change. Interestingly, messages containing explanations about the mechanisms of myopia development did not significantly enhance knowledge levels. This suggests prioritizing actionable content readily applicable by the target audience, such as the 20-20-20 rule [36]. Furthermore, while multimedia contents were initially expected to improve knowledge acquisition, SMS text messages without images or infographics proved to be sufficient promoting healthy behavior adoption. Finally, the inclusion of information within the message library to address common misconceptions about eye health enhanced the message credibility and fostered greater program satisfaction among participants. This research supports the phenomenon that people in today’s society are overloaded with information and find it difficult to recognize trustworthy sources. Our study found that the established reputation of WHO and YUHS positively influenced overall trust and engagement with the message and program. This highlights the importance of credible sources in promoting program participation and adherence.

### Limitations

This study has several limitations. First, recruitment challenges resulted in a smaller sample size than initially aimed. This could be attributed to parental concerns about a program targeting children. To mitigate such concerns in future studies, it is important to include a more comprehensive explanation of the program’s purpose and benefits while also acknowledging the possibility of participant withdrawal. Despite the limited sample size, this study used rigorous methodologies to ensure reliability. By incorporating WHO monitoring and assessment indicators in quantitative analysis and achieving saturation through consistent participant responses in qualitative analysis, the study provides a solid foundation for future large-scale implementation of the MyopiaEd program. Second, we only targeted parents who showed significant knowledge gains during the qualitative interviews. By focusing on this group, we could delve deeper into the key factors influencing how improved knowledge led to behavior change. Third, our study design had limitations in evaluating the long-term impact of the MyopiaEd program on myopia incidence and behavior change. As this was the first study to implement the WHO MyopiaEd program, the study adhered to the 6-month intervention and evaluation guidelines suggested by WHO. Future research should consider using a longitudinal design with a longer follow-up period to establish the sustainability of program effects.

### Conclusions

Our research suggests that the MyopiaEd program represents a promising intervention tool for mitigating the risk of myopia progression. The program’s demonstrated effectiveness in this study provides strong evidence to support its wider implementation and integration into national health policies. Moreover, the study underscores the pivotal role of digital interventions in combating the escalating prevalence of myopia in South Korea.

## Appendix

Multimedia Appendix 1 Summary of pretesting outcomes for the MyopiaEd message library.

Multimedia Appendix 2 Adapted MyopiaEd message library used for the intervention.

Multimedia Appendix 3 CHERRIES (Checklist for Reporting Results of Internet E-Surveys) summary.

Multimedia Appendix 4 Individual question scores from the parent knowledge assessment questionnaire.

Multimedia Appendix 5 Characteristics of participants in the qualitative research.

## Abbreviations

CHERRIES: Checklist for Reporting Results of Internet E-Surveys
COREQ: Consolidated Criteria for Reporting Qualitative Data
iCHECK-DH: Guidelines and Checklist for Reporting Digital Health Implementations
IRB: institutional review board
ITU: International Telecommunication Union
mHealth: mobile health
WHO: World Health Organization
YUHS: Yonsei University Health System

## Data Availability

The datasets generated or analyzed during this study are available from the corresponding author upon reasonable request.

## References

[1] Holden BA, Fricke TR, Wilson DA, Jong M, Naidoo KS, Sankaridurg P, Wong TY, Naduvilath TJ, Resnikoff S (2016). Global prevalence of myopia and high myopia and temporal trends from 2000 through 2050. Ophthalmology.

[2] Morgan IG, Ohno-Matsui K, Saw SM (2012). Myopia. Lancet.

[3] (2016). The impact of myopia and high myopia: report of the Joint World Health Organization–Brien Holden Vision Institute Global Scientific Meeting on Myopia, University of New South Wales, Sydney, Australia, 16–18 March 2015. World Health Organization.

[4] Bourne RRA, Stevens GA, White RA, Smith JL, Flaxman SR, Price H, Jonas JB, Keeffe J, Leasher J, Naidoo K, Pesudovs K, Resnikoff S, Taylor HR (2013). Causes of vision loss worldwide, 1990-2010: a systematic analysis. Lancet Glob Health.

[5] Tsai TH, Liu Y, Ma I, Su C, Lin C, Lin LL, Hsiao CK, Wang I (2021). Evolution of the prevalence of myopia among Taiwanese schoolchildren: a review of survey data from 1983 through 2017. Ophthalmology.

[6] Wang YX, Pan Z, Wang ZY, Li Z, Huang Y, Wang J, Zhang C, Li F, Jonas JB, Wong TY (2023). 25-year trend in myopia prevalence in Chinese school children and adolescents: a nationwide analysis from 1998 to 2022. Invest Ophthalmol Vis Sci.

[7] Ding BY, Shih Y, Lin LL, Hsiao CK, Wang I (2017). Myopia among schoolchildren in East Asia and Singapore. Surv Ophthalmol.

[8] Williams K, Hammond C (2019). High myopia and its risks. Community Eye Health.

[9] Pan CW, Dirani M, Cheng C, Wong T, Saw S (2015). The age-specific prevalence of myopia in Asia: a meta-analysis. Optom Vis Sci.

[10] Martínez-Albert N, Bueno-Gimeno I, Gené-Sampedro A (2023). Risk factors for myopia: a review. J Clin Med.

[11] Jones LA, Sinnott LT, Mutti DO, Mitchell GL, Moeschberger ML, Zadnik K (2007). Parental history of myopia, sports and outdoor activities, and future myopia. Invest Ophthalmol Vis Sci.

[12] Goldschmidt E, Jacobsen N (2014). Genetic and environmental effects on myopia development and progression. Eye (Lond).

[13] Huang HM, Chang DS, Wu PC (2015). The association between near work activities and myopia in children—a systematic review and meta-analysis. PLoS One.

[14] Saunders TJ, Vallance JK (2017). Screen time and health indicators among children and youth: current evidence, limitations and future directions. Appl Health Econ Health Policy.

[15] Tsai DC, Fang S, Huang N, Hsu C, Chen S, Chiu AW, Liu CJ (2016). Myopia development among young schoolchildren: the myopia investigation study in Taipei. Invest Ophthalmol Vis Sci.

[16] He X, Sankaridurg P, Wang J, Chen J, Naduvilath T, He M, Zhu Z, Li W, Morgan IG, Xiong S, Zhu J, Zou H, Rose KA, Zhang B, Weng R, Resnikoff S, Xu X (2022). Time outdoors in reducing myopia: a school-based cluster randomized trial with objective monitoring of outdoor time and light intensity. Ophthalmology.

[17] Xiong S, Sankaridurg P, Naduvilath T, Zang J, Zou H, Zhu J, Lv M, He X, Xu X (2017). Time spent in outdoor activities in relation to myopia prevention and control: a meta-analysis and systematic review. Acta Ophthalmol.

[18] Wu PC, Tsai C, Wu H, Yang Y, Kuo H (2013). Outdoor activity during class recess reduces myopia onset and progression in school children. Ophthalmology.

[19] Marcolino MS, Oliveira JAQ, D'Agostino M, Ribeiro AL, Alkmim MBM, Novillo-Ortiz D (2018). The impact of mHealth interventions: systematic review of systematic reviews. JMIR Mhealth Uhealth.

[20] (2023). Measuring digital development: facts and figures 2023. International Telecommunication Union.

[21] Ryu S (2012). Book review: mHealth: new horizons for health through mobile technologies: based on the findings of the second global survey on eHealth (Global Observatory for eHealth Series, Volume 3). Healthc Inform Res.

[22] Lester RT, Ritvo P, Mills EJ, Kariri A, Karanja S, Chung MH, Jack W, Habyarimana J, Sadatsafavi M, Najafzadeh M, Marra CA, Estambale B, Ngugi E, Ball TB, Thabane L, Gelmon LJ, Kimani J, Ackers M, Plummer FA (2010). Effects of a mobile phone short message service on antiretroviral treatment adherence in Kenya (WelTel Kenya1): a randomised trial. Lancet.

[23] Donner J (2008). Research approaches to mobile use in the developing world: a review of the literature. Inform Soc.

[24] Gopinathan P, Kaur J, Joshi S, Prasad VM, Pujari S, Panda P, Murthy P (2018). Self-reported quit rates and quit attempts among subscribers of a mobile text messaging-based tobacco cessation programme in India. BMJ Innov.

[25] Ramachandran A, Kumar R, Nanditha A, Raghavan A, Snehalatha C, Krishnamoorthy S, Joshi P, Tesfaye F (2018). mDiabetes initiative using text messages to improve lifestyle and health-seeking behaviour in India. BMJ Innov.

[26] Wargny M, Kleinebreil L, Diop SN, Ndour-Mbaye M, Ba M, Balkau B, Simon D (2018). SMS-based intervention in type 2 diabetes: clinical trial in Senegal. BMJ Innov.

[27] Keel S, Govender-Poonsamy P, Cieza A, Faal H, Flitcroft I, Gifford K, He M, Khandekar R, Naidoo K, Oerding M, Ohno-Matsui K, Mariotti S, Wildsoet C, Wolffsohn JS, Wong TY, Yoon S, Mueller A, Dobson R (2022). The WHO-ITU MyopiaEd programme: a digital message programme targeting education on myopia and its prevention. Front Public Health.

[28] Pärssinen O, Lyyra AL (1993). Myopia and myopic progression among schoolchildren: a three-year follow-up study. Invest Ophthalmol Vis Sci.

[29] Lee DC, Lee SY, Kim YC (2018). An epidemiological study of the risk factors associated with myopia in young adult men in Korea. Sci Rep.

[30] Poushter J, Gubbala S, Austin S (2024). 8 charts on technology use around the world. Pew Research Center.

[31] Tong A, Sainsbury P, Craig J (2007). Consolidated Criteria for Reporting Qualitative Research (COREQ): a 32-item checklist for interviews and focus groups. Int J Qual Health Care.

[32] Perrin Franck C, Babington-Ashaye A, Dietrich D, Bediang G, Veltsos P, Gupta PP, Juech C, Kadam R, Collin M, Setian L, Serrano Pons J, Kwankam SY, Garrette B, Barbe S, Bagayoko CO, Mehl G, Lovis C, Geissbuhler A (2023). iCHECK-DH: Guidelines and Checklist for the Reporting on Digital Health Implementations. J Med Internet Res.

[33] (2022). Be He@lthy, Be Mobile: a toolkit on how to implement MyopiaEd. World Health Organization and International Telecommunication Union.

[34] Kim H, Seo JS, Yoo W, Kim G, Kim RB, Chae JE, Chung I, Seo S, Kim SJ (2020). Factors associated with myopia in Korean children: Korea National Health and Nutrition Examination Survey 2016-2017 (KNHANES VII). BMC Ophthalmol.

[35] (2024). Share of mobile messenger users who use KakaoTalk in South Korea as of December 2023, by age group. Statista.

[36] (2022). MyopiaEd message libraries Be He@lthy, Be Mobile: a toolkit on how to implement MyopiaEd, Web Annex, MyopiaEd message libraries. World Health Organization and International Telecommunication Union.

[37] Eysenbach G (2004). Improving the quality of web surveys: the Checklist for Reporting Results of Internet E-Surveys (CHERRIES). J Med Internet Res.

[38] McCrann S, Flitcroft I, Lalor K, Butler J, Bush A, Loughman J (2018). Parental attitudes to myopia: a key agent of change for myopia control?. Ophthalmic Physiol Opt.

[39] Chua SYL, Sabanayagam C, Cheung Y, Chia A, Valenzuela RK, Tan D, Wong T, Cheng C, Saw S (2016). Age of onset of myopia predicts risk of high myopia in later childhood in myopic singapore children. Ophthalmic Physiol Opt.

[40] Li Q, Guo L, Zhang J, Zhao F, Hu Y, Guo Y, Du X, Zhang S, Yang X, Lu C (2021). Effect of school-based family health education via social media on children's myopia and parents' awareness: a randomized clinical trial. JAMA Ophthalmol.

[41] Kim JS, Bang H (2016). Education fever: Korean parents’ aspirations for their children’s schooling and future career. Pedagogy Cult Soc.

[42] (2023). MSIT announces 2022 survey result on digital divide: smartphone overdependence survey. Ministry of Science and ICT.

[43] Gong N, Wu X, Zhang Y, Meng Y, Sun S, Xie J, Yao L, Cheng Y, Zhang M (2022). Barriers to family intervention to promote child and adolescent vision health: a qualitative study based on community practice in China. J Pediatr Nurs.

[44] (2020). Household chores time by marital status and dual-earner status. Statistics Korea.

