# Supplementary material for: Evaluating the Effectiveness and Scalability of the World Health Organization MyopiaEd Digital Intervention: Mixed Methods Study
Source: JMIR Public Health Surveill. 2024 Dec 16;10:e66052. doi: 10.2196/66052 (PMC11686028; doi:10.2196/66052)
Supplement: Multimedia Appendix 1 [file publichealth_v10i1e66052_app1.pdf]

Pre-testing

Participants

The target population for the pre-testing phase was parents of children aged 7-8 years old. Three parents participated in the pre-testing. Prior to enrollment, all participants provided written informed consent and agreed to be involved in this study. All participants resided in Gwangju Metropolitan City, South Korea and were female.

Intervention and evaluation

Participants received 14 core MyopiaEd messages selected from the library in Korean over a two-week period. They were informed to provide feedback on both the message content and the overall program design as part of the pre-testing phase.

To assess participant feedback, the pre-testing process utilized two primary methods: Focus Group Interviews (FGIs) and telephone interviews. Firstly, FGI sessions were conducted in person with two parents of children. A trained interviewer facilitated each session which followed a guideline and lasted between 60 and 90 minutes.[1] All sessions were voice-recorded and subsequently transcribed using speech-to-text software. Secondly, a telephone interview was conducted with one parent who was unable to participate in an FGI session. This interview served to gather their feedback as well.

Key Feedback received

The MyopiaEd program received positive feedback from participants, with many showing strong interest by wanting to sign up for the full program. They mentioned the messages' clarity and alignment with program goals, particularly highlighting the ease of understanding the information. However, there were suggestions for improvement. While both groups acknowledged the messages' overall helpfulness, they felt it lacked depth and sometimes repeated general information what they already knew. The main feedback was regarding incorporating more visual elements, diversifying message topics, strengthening the information with evidence-based support, and providing practical guidelines for participants to follow.

1. Message content visualization
- Utilize multimedia content, such as photographs, illustrations, and comics, instead of plain text to enhance participant interest, program engagement, and message delivery.
- Provide activity sheets and educational materials that parents and children can work on collaboratively to encourage eye health checkups.

2. Avoid repetition and diversify content

---

[1] Krueger, R. A. (2002, October). Designing and Conducting Focus Group Interviews. University of Minnesota.

- Refrain from repetition in message content to avoid participants feeling like they are receiving advertisements, which can lead to decreased message engagement and program interest.
- Diversify message topics to maintain participant interest.

3. Strengthen content with professional and medical knowledge
- Prioritize the quality of message content by incorporating medical evidence, even if this results in longer messages, rather than providing abstract direction.
- Utilize numerical data and statistics to enhance the persuasiveness of the messages.

4. Provide clear guidelines and practical information
- Establish specific goals and guidelines to encourage active participation.
- Provide practical information regarding eye examinations, including their associated costs, and applicable health insurance coverage.

Specific feedback from the pre-testing included:

- Parents mentioned that their children often spend a lot of free time indoors due to after-school activities. They suggested the program address these situations and provide practical tips for managing screen time during these periods.
- Parents indicated that additional images or illustrations related to the messages would be helpful when sharing the information with their children.
- They requested more in-depth information about the science and evidence behind the advice to make it more persuasive. Even if the messages became longer, they preferred detailed and professional information that saved them from having to research the topics themselves after receiving the message. E.g. specific eye abnormality symptoms and age-appropriate eye examination types.
- Parents wanted the messages to include clear and direct advice for their children that could be easily shared with them.
- Parents suggested including specific examples of what to look at in the distance (e.g., a tree outside) instead of just numbers for the 20-20-20 rule.
- Parents identified a common misconception about carrots and blueberries improving eyesight. They felt this information was valuable to share with others who were not included in the program.
- Parents requested more specific clinical evidence from eye health professionals to address misconceptions about wearing prescribed glasses.

Feedback on the frequency and channel of messaging:

1. Timing
- Parents preferred receiving messages at times when they were likely to be with their children, such as after school or in the evenings. This allows them to discuss the message content with their children directly.

2. Messaging Channel

- All participants expressed satisfaction with the messaging app due to its convenience. The app allows them to confirm receipt of messages and archive them for future reference.

Key changes made to the message library as a result of the pre-testing

- Incorporated evidence-based visual content to enhance readability and user engagement. This development was achieved through a partnership with Weknew, a communication and design agency specializing in the healthcare sector.
- Diversified the topics of general information to maintain participant interest, including trending eye health issues like orthokeratology (Ortho-K) and blue light-blocking glasses.
- Addressed myopia with clinical and evidence-based knowledge from an eye health professional to enhance the persuasiveness of the messages.
- Established specific goals and guidelines that could be easily adapted to daily lives, including providing specific examples of what to look at in the distance (e.g., looking at a tree outside).
