# Supplementary material for: Evaluating the Effectiveness and Scalability of the World Health Organization MyopiaEd Digital Intervention: Mixed Methods Study
Source: JMIR Public Health Surveill. 2024 Dec 16;10:e66052. doi: 10.2196/66052 (PMC11686028; doi:10.2196/66052)
Supplement: Multimedia Appendix 2 [file publichealth_v10i1e66052_app2.pdf]

Week	ID	Theme	Message (English Translation)
0		Admin	[hi] [parent's name]. Welcome to the MyopiaEd program. We will be sending you messages about myopia to care for [child's name]'s eye health for the next 6 months.
1	PM1	General knowledge about myopia	[parent's name], do you know what myopia is? Myopia, one of the eye diseases, means that a person can see clearly up close, but not well far away.<br><br>Do you ever wonder how myopia affects your child's vision? Why not check out our "How the Myopic Eye Sees" with your child? If [child's name] sees distant objects blurry like the photos, it might be time for an eye exam. An eye health professional is here to support and help you.<br><br>Check it out: How the Myopic Eye Sees [website link]
	PM2	Myopia misconceptions	[Useful Tips for Your Eye Health #1]<br>Q. Is it true that eating carrots or blueberries improves my vision?<br><br>A. The World Health Organization (WHO) confirms it can be part of a healthy diet that supports good eye health, but it won't directly improve your vision. Here's the key: glasses prescribed by an eye doctor are the best way to correct blurry vision. There are simple daily practices to keep your eyes healthy too!
2	PM3	Behaviour change/ lifestyle factors	[parent's name], it's not easy to know if [child's name] is having trouble in eyes. Children might not always realize or tell you if their eyesight changes.<br>Here's a tip: watch for squinting or rubbing their eyes when looking far away. Lots of kids who sit close to the TV might have developing myopia.<br>Your love and attentiveness to [child's name]'s behavior is the first step in managing their eye health!
	PM4	General knowledge about myopia	[parent's name], today is World Sight Day! If you are curious about why and how it was created, check out our campaign blog: [website link]<br><br>Why don't you share a #LOVE_YOUR_EYES message with your family, friends, and people around you celebrating the World Sight Day?<br>The Love Your Eyes campaign will be held at the [School name]. Please pay close attention to the update on the campaign blog!

3	PM5	General knowledge about myopia	[parent's name], over 20% of the world population has myopia, and even more wear glasses. Myopia has different causes, but the main cause is 'prolonged near work activities'.<br>[parent's name] can help slow down the progression of [child's name]'s myopia.<br><br>If you want to learn more about the risk factors for myopia, visit our campaign blog!<br>[website link]
	PM6	Behaviour change/ lifestyle factors	[parent's name], spending time outdoors can actually help slow down its progression. Aim for at least 90 minutes of playtime in the sun every day.<br><br>On a nice day like today, why don't you take a walk in the sun with [child's name]?
4	PM7	Behaviour change/ lifestyle factors	[parent's name], how about making eye care fun for [child's name]? Watch the video below together<br><br>[video link]
	PM8	Myopia misconceptions	[Useful Tips for Your Eye Health #2]<br>Q. Does wearing glasses make my eyesight worse?<br>A. That's not true. Glasses can help not only see clearly but also prevent eye strain.<br><br>If [child's name] has myopia, wearing glasses is a great way to see clearly and potentially slow the progression. If you're curious about other ways to manage myopia, check out our campaign blog!<br>[website link]
5	PM9	General knowledge about myopia	[parent's name], in addition to glasses, there are ways to correct myopia, such as contact lenses and atropine eye drops.<br><br>To find the best approach for [child's name], schedule an appointment with eye health professional. They can discuss treatment options and recommend any precautions you should keep in mind.
	PM10	Compliance with correction	Did you know that untreated childhood myopia can raise the risk of other eye problems later in life? High myopia that develops in childhood can also increase the chance of complications leading to blindness in adulthood.<br><br>[parent's name], for [child's name]'s eye health, you have to start taking care of it from now on!<br>Check it out: Why's high myopia a concern? [website link]

6	PM11	Behaviour change/ lifestyle factors	Healthy eyes are fundamental for various activities like reading and exercising. If your vision isn't clear, it can be difficult to carry out your daily tasks effectively.<br><br>Try asking [child's name] "Are your eyes feeling alright? Can you see the whiteboard clearly?". Keeping an eye on their vision is important!
	PM12	Need for eye exams	[Useful Tips for Your Eye Health #3]<br>Q. Where can I get an eye examination?<br>A. Eye examination might seem quick and easy, but it's a key part of the healthcare. Get your eyes checked by an eye health professional!
7	PM13	Need for eye exams	[parent's name], ever feel unsure about what eye exams [child's name] might need?<br><br>Our campaign blog has all the information you need on eye exams by age group, including what's best for [child's name]. Why not schedule a family eye exam together?<br>[website link]
	PM14	Myopia misconceptions	[Useful Tips for Your Eye Health #4]<br>Q. How many blueberries should I eat to improve my eye health?<br>A. Carrots and blueberries...<br>[parent's name], if you're curious about the World Health Organization (WHO)'s answer to this question, please check out our campaign blog!<br>[website link]
8	PM15	Compliance with correction	[parent's name], did you know wearing glasses might do more than just help [child's name] see clearly?<br>Some studies reported it could even boost their learning! Check out our blog for more on how myopia correction can impact academic achievement and motivation.
	PM16	The need for eye exams	Everyone’s eyes are different.<br>[parent's name], for your precious eye health, regular eye exams and following an eye health professional's advice are key.
9	PM17	General knowledge about myopia	[Useful Tips for Your Eye Health #5]<br>Q. Do I need to wear blue light-blocking glasses?<br>A. Many people wear blue light-blocking glasses. [parent's name], wondering about blue light and if those glasses really work? Please visit our blog below!<br>[website link]

	PM18	Behaviour change/ lifestyle factors	[parent's name], hope you and [child's name] are having a fantastic weekend!<br>Let's practice useful tips with [child's name] to avoid blue light that can disrupt sleep and start the week feeling refreshed!<br>No blue light-blocking glasses needed – just some lifestyle changes for a better night's rest!
10	PM19	Behaviour change/ lifestyle factors	[Parent's name], staring at screens all day can be rough on your eyes. Let's try some easy tips together, like the 20-20-20 rule to relieve eye strain.<br>Check it out: Tips for eye health you don't want to miss [website link]
	PM20	Increasing engagement	[parent's name], do you have any question about [child's name]'s myopia?<br>Kindly send a message to our KakaoTalk channel so that we can answer your questions.<br>Look forward to hearing from you!
11	PM21	General knowledge about myopia	[parent's name], winter's here! Cold weather brings more fine dust! This air pollution increases the risk of respiratory infections, but did you know it can irritate the eyes too?<br><br>Check out our blog to eye health and fine dust to learn more. [website link]
	PM22	General knowledge about myopia	[parent's name], there's an air quality alert today in Gwangju! Fine dust levels are up to 'Very Bad' with severe yellow dust.<br>On days with severe fine dust, there's a higher chance of eye problems like allergic conjunctivitis and dry eye.<br><br>Here's the good news: we have 3 easy steps you can take to protect your precious eyes! [website link]
12	PM23	The need for eye exams	[parent's name], ever wonder why eye exams are important for [child's name]?<br><br>You can't measure eyesight without examination, so regular checkups are key to monitoring eye conditions.<br><br>If you're curious about the timing and method of eye examination for infants, please check out our campaign blog! Check it out: When is the Critical Period for Vision? [website link]
	PM24	The need for eye exams	[parent's name], getting eye exams every 6 months is just as important for [child's name] as dental checkups!<br><br>As the year ends, why not schedule those eye exams you've been planning to get?

13	PM25	The Need for eye exams	[parent's name], it's not easy to know if [child's name] is having trouble with his/her eyes because children are not good at expressing their symptoms.<br><br>Watch for signs like squinting, rubbing their eyes, or sitting too close to the TV. If you notice any signs, visit an eye health professional for a checkup. This is the first step to protecting your child's eye health.<br><br>Want to learn more about these signs? Check out our blog for details! [website link]
	PM26	Behaviour change/ lifestyle factors	[Q&A #1] Q. Reading in the dark vs. Looking at electronic devices closely - which is worse?<br>A. Both are near work activities that require focus and can strain your eyes! Spending a long time focusing on close-up objects can make it harder to switch your focus between near and far distances, contributing to myopia development.<br><br>However, many people perceive that watching videos is worse. This might be because videos are more engaging than books, leading to longer screen time.<br><br>※ The Q&A section is organized based on the questions sent by the participants.
14	PM27	The Need for eye exams	Let's welcome the new year by setting some eye health goals for [child's name]!<br><br>Here's a great idea: add "scheduling an eye exam to check [child's name]'s eye health and consulting with an eye health professional" to your list of goals.'
	PM28	Behaviour change/ lifestyle factors	[Q&A #2] Q. I heard reading can worsen myopia. Should I limit my child's reading?<br><br>A. That's a common concern! Near activities like reading or using smartphones can indeed affect myopia progression. But here's the good news: there's no need to stop reading books! The key is to develop good reading habits.<br><br>Check out our blog for tips about how to read books without straining eyes!<br><br>Check it out: Healthy Reading Habits [website link]
15	PM29	General knowledge about myopia	[Useful Tips for Your Eye Health #6]<br><br>[parent's name], are you considering vision correction surgery for [child's name] in the future? Myopia management can be a great long-term strategy!

			While vision correction surgery can be an option, high myopia always carries a high risk.<br><br>Please check the campaign blog for information on the side effects and the recommended timing of surgery, as advised by eye health professionals!
	PM30	Behaviour change/ lifestyle factors	[Q&A #3] Reading by Living Room Lights vs. Desk Lamp?<br><br>A. Ideal illuminance (light intensity) for reading is between 400 and 500 lux.<br><br>The distance from the lighting in the living room to the book might be too far for reading. So, if you consider illuminance, reading at a desk with a good reading lamp is generally the better option for consistent and eye-friendly lighting.<br><br>However, if you prefer to read in the living room, using an extra lamp near your book can help keep your eyes healthy!<br><br>※ The Q&A section is organized based on the questions sent by the participants.
16	PM31	General knowledge about myopia	[Q&A #4] Q. From what age is it acceptable for children to wear lenses?<br><br>A. Parental guidance and support play a crucial role in managing a child's Ortho-K treatment. While children should be able to handle lens hygiene and care independently for regular soft lenses, they are not usually recommended for teenagers due to the higher risk of infections compared to glasses. Contact lenses are generally a better fit for adults who can ensure proper self-care.<br><br>※ The Q&A section is organized based on the questions sent by the participants.
	PM32	General knowledge about myopia	[parent's name], have you ever wondered how ortho-k lenses work differently than regular ones? Check out our blog for information about the science behind them, potential downsides, and wearing duration!<br>[website link]<br><br>Before using Ortho-K, it is necessary to have a sufficient understanding of how to wear and manage lenses through adequate consultation with an eye health professional.

17	PM33	Behaviour change/ lifestyle factors	[parent's name], did you practice healthy habits to prevent myopia with [child's name] today? Remember, healthy eyes mean healthy habits! Here are a few quick tips: 20-20-20 rule, Arm's length activities, and 90 minutes of outdoor activity.
	PM34	The need for eye exams	[parent's name], growing kids like [child's name] can experience faster vision changes, especially during growth spurts!<br>Regular eye exams every 6 months is the key. Check for changes in vision constantly and make sure to wear glasses that fit for their growing eyes.<br><br>Why not schedule [child's name]'s eye exam during the upcoming vacation?
18	PM35	General knowledge about myopia	Dry eye syndrome is an inevitable disease for modern people living in the digital age.<br>Winter dryness, like these days, can certainly make things worse, but there are ways to keep your eyes feeling comfortable.<br><br>Here's a blog post to learn more: [website link]
	PM36	General knowledge about myopia	[Useful Tips for Your Eye Health #7]<br>Q. I am constantly taking eye health supplements; will they be helpful?<br><br>A. [BLANK] is important for overall eye health!<br>Here's why: Studies have shown that a lack of essential nutrients can contribute to eye problems.<br><br>Curious to know what is blank? Check out our campaign blog! [website link]
19	PM37	The need for eye exams	[parent's name], winter break is almost here - the perfect time to schedule that eye exam for [child's name]!<br><br>Visit an eye health professional can help ensure [child's name]'s vision is developing well and catch any potential issues like myopia or astigmatism early on.
20	PM38	Myopia misconceptions	[parent's name], are you worried because [child's name]'s eyes keep getting worse even with glasses?<br><br>Wearing glasses doesn't worsen vision. Discover the reasons behind changes in vision and how glasses can help on our campaign blog!<br>[website link]<br><br>Habits and eye health care during adolescence determine lifelong vision.

21	PM39	General knowledge about myopia	[Q&A #5] Q. Can I use artificial tears frequently?<br><br>A. Absolutely. Here's a tip: Since your eyes can only absorb a small amount at a time, one drop per eye is sufficient. There's no need to reapply more often than once every hour. For even greater safety, consider using preservative-free artificial tears!<br><br>※ The Q&A section is organized based on the questions sent by the participants.
22	PM40	General knowledge about myopia	[parent's name], people with severe high myopia are at an increased risk of developing sight-threatening complications, typically later in life. Learn about one of these complications, retinal detachment, in the blog.<br>[website link]<br><br>It is crucial to prevent [child's name]'s myopia from progressing to high myopia.
23	PM41	Behaviour change/ lifestyle factors	[parent's name], does [child's name] enjoy reading books or using a smartphone? Spending lots of time doing near activities can give burden to his/her eyes.<br><br>The good news? We can create an eye-friendly environment together! Let's help [child's name] develop healthy habits to keep their peepers happy. Even small changes can make a big difference!<br>[website link]
24	PM42	The need for eye exams	[parent's name], When was [child's name]'s last eye check?<br>Timely treatment is key for preventing permanent vision problems in children with myopia during their growth period.<br>Regular eye exams every 6 months are recommended.
		Admin	[hi] [parent's name]. You have now finished the MyopiaEd message program. Thanks for taking part of the program.<br>We will contact you soon with a post-survey guide.
