# Supplementary material for: Evaluating the Effectiveness and Scalability of the World Health Organization MyopiaEd Digital Intervention: Mixed Methods Study
Source: JMIR Public Health Surveill. 2024 Dec 16;10:e66052. doi: 10.2196/66052 (PMC11686028; doi:10.2196/66052)
Supplement: Multimedia Appendix 3 [file publichealth_v10i1e66052_app3.pdf]

Checklist Item		Explanation
**Describe survey design**	Describe target population, sample frame. Is the sample a convenience sample? (In “open” surveys this is most likely.)	The target population for the survey was parents of 7- and 8-year-old children.
**IRB approval**	Mention whether the study has been approved by an IRB.	The study was approved by the Institutional Review Board (IRB), Severance Hospital, Yonsei University, Seoul, Korea (IRB Approval Number. 4-2022-0798).
**Informed consent**	Describe the informed consent process. Where were the participants told the length of time of the survey, which data were stored and where and for how long, who the investigator was, and the purpose of the study?	Prior to submitting their responses, participants were provided with a one-page introductory note outlining the research purpose, investigator identity, estimated survey duration, and data storage methods.
**Data protection**	If any personal information was collected or stored, describe what mechanisms were used to protect unauthorized access.	All personally identifiable information collected from study participants was anonymized prior to statistical analysis. This ensures that no individual participant can be identified from the research data. Participant information was stored in a separate, coded file under the principal investigator's supervision, preventing any linkage between individual participants and their data.
**Development and testing**	State how the survey was developed, including whether the usability and technical functionality of the electronic questionnaire had been tested before fielding the questionnaire.	The survey was created using Google Forms. Prior to deployment, two independent researchers conducted a thorough technical review of the electronic questionnaire.
**Open survey versus closed survey**	An “open survey” is a survey open for each visitor of a site, while a closed survey is only open to a sample which the investigator knows (password-protected survey).	Once participant recruitment was complete, the survey link was sent to all enrolled participants.
**Contact mode**	Indicate whether or not the initial contact with the potential participants was made on the Internet. (Investigators may also send out questionnaires by mail and allow for Web-based data entry.)	Enrolled participants were initially contacted via KakaoTalk, messaging app.
**Advertising the survey**	How/where was the survey announced or advertised? Some examples are offline media (newspapers), or online (mailing lists – If yes, which ones?) or banner ads (Where were these banner ads posted and what did	Enrolled participants received a survey invitation via KakaoTalk, messaging app.

	they look like?). It is important to know the wording of the announcement as it will heavily influence who chooses to participate. Ideally the survey announcement should be published as an appendix.	The invitation included a link to a Google Forms survey and stated: Welcome to the MyopiaEd program. To register for the program, please complete the pre-survey. Thank you for taking the time to complete the survey by the deadline.
**Web/E-mail**	State the type of e-survey (eg, one posted on a Web site, or one sent out through e-mail). If it is an e-mail survey, were the responses entered manually into a database, or was there an automatic method for capturing responses?	Google Forms
**Context**	Describe the Web site (for mailing list/newsgroup) in which the survey was posted. What is the Web site about, who is visiting it, what are visitors normally looking for? Discuss to what degree the content of the Web site could pre-select the sample or influence the results. For example, a survey about vaccination on a anti-immunization Web site will have different results from a Web survey conducted on a government Web site	Google Forms
**Mandatory/voluntary**	Was it a mandatory survey to be filled in by every visitor who wanted to enter the Web site, or was it a voluntary survey?	Voluntary survey
**Incentives**	Were any incentives offered (eg, monetary, prizes, or non-monetary incentives such as an offer to provide the survey results)?	As compensation for completing the pre- and post-surveys, participants received a 100,000 KRW (76.32 USD) gift certificate for an optical store.
**Time/Date**	In what timeframe were the data collected?	1. The pre-survey was conducted in Sep 2021.<br>2. The post-survey was conducted from Feb to March 2022.
**Randomization of items or questionnaires**	To prevent biases items can be randomized or alternated.	No items or questionnaires were randomized.
**Adaptive questioning**	Use adaptive questioning (certain items, or only conditionally displayed based on responses to other items) to reduce number and complexity of the questions.	Adaptive questioning was used.

**Number of Items**	What was the number of questionnaire items per page? The number of items is an important factor for the completion rate.	1. The pre-survey consisted of 30 questions, divided as follows:<br>- Demographic questions: 4<br>- Knowledge measurement questions: 16<br>- Behavior questions: 10<br><br>2. The post-survey consisted of 38 questions, divided as follows:<br>- Demographic questions: 4<br>- Self-reported change in knowledge/behavior measurement questions: 8<br>- Knowledge measurement questions: 16<br>- Behavior questions: 6<br>- Overall feedback for the program: 4<br><br>The number of items per page varied from 4 to 16.
**Number of screens (pages)**	Over how many pages was the questionnaire distributed? The number of items is an important factor for the completion rate.	1. Pre-survey: 3 pages<br>2. Post-survey: 4 pages
**Completeness check**	It is technically possible to do consistency or completeness checks before the questionnaire is submitted. Was this done, and if "yes", how (usually JAVAScript)? An alternative is to check for completeness after the questionnaire has been submitted (and highlight mandatory items). If this has been done, it should be reported. All items should provide a non-response option such as "not applicable" or "rather not say", and selection of one response option should be enforced.	Participants were required to complete all mandatory questions before submitting their responses. A 'Don't know' option was provided for optional questions to avoid forcing responses.
**Review step**	State whether respondents were able to review and change their answers (eg, through a Back button or a Review step which displays a summary of the responses and asks the respondents if they are correct).	Respondents were able to review and change their answers before submission.
**Unique site visitor**	If you provide view rates or participation rates, you need to define how you determined a unique visitor. There are different techniques available, based on IP addresses or cookies or both.	Unique site/survey visitors were not tracked. Only enrolled participants received the survey form link.

**View rate (Ratio of unique survey visitors/unique site visitors)**	Requires counting unique visitors to the first page of the survey, divided by the number of unique site visitors (not page views!). It is not unusual to have view rates of less than 0.1 % if the survey is voluntary.	N/A
**Participation rate (Ratio of unique visitors who agreed to participate/unique first survey page visitors)**	Count the unique number of people who filled in the first survey page (or agreed to participate, for example by checking a checkbox), divided by visitors who visit the first page of the survey (or the informed consents page, if present). This can also be called “recruitment” rate.	N/A
**Completion rate (Ratio of users who finished the survey/users who agreed to participate)**	The number of people submitting the last questionnaire page, divided by the number of people who agreed to participate (or submitted the first survey page). This is only relevant if there is a separate “informed consent” page or if the survey goes over several pages. This is a measure for attrition. Note that “completion” can involve leaving questionnaire items blank. This is not a measure for how completely questionnaires were filled in. (If you need a measure for this, use the word “completeness rate”.)	133 of 184 participants completed the survey, for a completion rate of 72.3 %.
**Cookies used**	Indicate whether cookies were used to assign a unique user identifier to each client computer. If so, mention the page on which the cookie was set and read, and how long the cookie was valid. Were duplicate entries avoided by preventing users access to the survey twice; or were duplicate database entries having the same user ID eliminated before analysis? In the latter case, which entries were kept for analysis (eg, the first entry or the most recent)?	No cookies were used.
**IP check**	Indicate whether the IP address of the client computer was used to identify potential duplicate entries from the same user. If so, mention the period of time for which no two entries from the same IP address were allowed (eg, 24 hours). Were duplicate entries avoided by preventing users with the same IP address access to	No IP tracking was used.

	the survey twice; or were duplicate database entries having the same IP address within a given period of time eliminated before analysis? If the latter, which entries were kept for analysis (eg, the first entry or the most recent)?	
**Log file analysis**	Indicate whether other techniques to analyze the log file for identification of multiple entries were used. If so, please describe.	No log file analysis was used for identification of multiple entries.
**Registration**	In “closed” (non-open) surveys, users need to login first and it is easier to prevent duplicate entries from the same user. Describe how this was done. For example, was the survey never displayed a second time once the user had filled it in, or was the username stored together with the survey results and later eliminated? If the latter, which entries were kept for analysis (eg, the first entry or the most recent)?	Participants’ names were stored temporarily with survey responses to enable the detection of duplicate submissions. However, all personally identifiable information including names were removed from the data before analysis to ensure participant anonymity.<br>There were no duplicate answers.
**Handling of incomplete questionnaires**	Were only completed questionnaires analyzed? Were questionnaires which terminated early (where, for example, users did not go through all questionnaire pages) also analyzed?	Only completed questionnaires were analyzed.
**Questionnaires submitted with an atypical timestamp**	Some investigators may measure the time people needed to fill in a questionnaire and exclude questionnaires that were submitted too soon. Specify the timeframe that was used as a cut-off point, and describe how this point was determined.	N/A
**Statistical correction**	Indicate whether any methods such as weighting of items or propensity scores have been used to adjust for the non-representative sample; if so, please describe the methods.	N/A

This checklist has been modified from Eysenbach G. Improving the quality of Web surveys: the Checklist for Reporting Results of Internet E-Surveys (CHERRIES). J Med Internet Res. 2004 Sep 29;6(3):e34. doi: 10.2196/jmir.6.3.e34. Erratum in: doi:10.2196/jmir.2042. PMID: 15471760; PMCID: PMC1550605.
