# Supplementary material for: Evaluating the Effectiveness and Scalability of the World Health Organization MyopiaEd Digital Intervention: Mixed Methods Study
Source: JMIR Public Health Surveill. 2024 Dec 16;10:e66052. doi: 10.2196/66052 (PMC11686028; doi:10.2196/66052)
Supplement: Multimedia Appendix 4 [file publichealth_v10i1e66052_app4.pdf]

No.	Questionnaire [a]	Parents of myopic children (n=60)			Parents of non-myopic children (n=73)		
		**Pre-survey, mean**	**Post-survey, mean**	***P* value**	**Pre-survey, mean**	**Post-survey, mean**	***P* value**
1	Myopia, also known as nearsightedness, is a condition where nearby objects can be seen clearly, but distant objects appear blurry.	91.67	91.67	1.00	84.93	93.15	0.09
2	Myopia can develop during growth, and its progression rate increases rapidly during this period.	96.67	96.67	1.00	91.78	91.78	1.00
3	As myopia progresses, habits like squinting and eye strain may develop, accompanied by headaches.	91.67	98.33	0.129	93.15	97.26	0.23
4	Genetic factors have a greater influence on the development and progression of myopia than environmental factors.	40.00	71.67	P<.001	52.05	75.34	0.002
5	Myopia starts at -0.5 diopters (D), and severe myopia is considered at -5.00D or -6.00D and below.	43.33	60.00	0.027	27.40	67.12	P<.001
6	Myopia is not caused by reading books or staring at TV monitors from close distances.	46.67	46.67	1.00	56.16	57.53	0.866
7	The progression of myopia is a natural phenomenon, and there are no specific preventive measures.	80.00	90.00	0.04	87.67	94.52	0.178
8	There is no correlation between reducing screen time and preventing eye diseases.	3.33	3.33	1.00	4.11	5.48	0.777
9	Increasing outdoor activities can help prevent myopia.	83.33	100.00	0.002	83.56	97.26	0.008
10	Wearing glasses helps vision correction.	81.67	96.67	0.01	80.82	94.52	0.004
11	When people wear glasses, their myopia may worsen initially.	83.33	95.00	0.02	87.67	95.89	0.066
12	Continuous use of glasses can increase dependence on them and consequently affect visual impairment.	78.33	93.33	0.014	79.45	86.30	0.208
13	Wearing glasses with incorrect prescriptions may worsen myopia.	91.67	91.67	1.00	94.52	95.89	0.77
14	Regular eye check-ups help in managing eye health and improving vision.	98.33	100.00	1.00	98.63	100.00	1.00

15	Unless there are specific symptoms, there is no need for regular eye check-ups.	95.00	100.00	0.149	90.41	98.63	0.04
16	If a someone is diagnosed with myopia and starts wearing glasses, regular monitoring of vision and glasses condition is necessary.	98.33	100.00	1.00	98.63	98.63	1.00

[a] Reverse coded: Question 4, 6, 7, 11, 12, 16
