# Supplementary material for: Evaluating the Effectiveness and Scalability of the World Health Organization MyopiaEd Digital Intervention: Mixed Methods Study
Source: JMIR Public Health Surveill. 2024 Dec 16;10:e66052. doi: 10.2196/66052 (PMC11686028; doi:10.2196/66052)
Supplement: Multimedia Appendix 5 [file publichealth_v10i1e66052_app5.pdf]

ID	Children's myopia	Change in knowledge score of parents	Eye exam uptake of children	Vision correction of children		Change in time spent outdoors of children	Change in time spent on near-work activities of children
				Pre-survey	Post-survey		
Parent 1	No	Increased	Yes	No	Yes	Increased	Decreased
Parent 2	No	Increased	Yes	No corrections are needed.		Increased	Increased
Parent 3	Yes	Increased	Yes	Hesitant about wearing eyeglasses, planning to decide in the follow-up exam.		Increased	Decreased
Parent 4	No	Increased	Yes	No	Yes	Increased	Increased
Parent 5	Yes	Increased	Yes	No	Yes	Decreased	Increased
Parent 6	No	Increased	Yes	No	Yes	Decreased	Decreased
Parent 7	Yes	Increased	Yes	No	Yes	Decreased	Increased
